# A new deep-reef scorpionfish (Teleostei, Scorpaenidae, *Scorpaenodes*) from the southern Caribbean with comments on depth distributions and relationships of western Atlantic members of the genus

**DOI:** 10.3897/zookeys.606.8590

**Published:** 2016-07-21

**Authors:** Carole C. Baldwin, Diane E. Pitassy, D. Ross Robertson

**Affiliations:** 1 Department of Vertebrate Zoology, National Museum of Natural History, Smithsonian Institution, Washington, DC 20560; 2 Smithsonian Tropical Research Institute, Balboa, Republic of Panamá

**Keywords:** Manned submersible, Smithsonian Deep Reef Observation Project (DROP), Substation Curaçao, ocean exploration, integrative taxonomy, phylogeny

## Abstract

A new species of scorpionfish, *Scorpaenodes barrybrowni* Pitassy & Baldwin, **sp. n.** which is described, was collected during submersible diving in the southern Caribbean as part of the Smithsonian’s Deep Reef Observation Project (DROP). It differs from the other two western Atlantic species of the genus, *Scorpaenodes caribbaeus* and *Scorpaenodes tredecimspinosus*, in various features, including its color pattern, having an incomplete lateral line comprising 8–10 pored scales, tending to be more elongate, usually having the 11^th^–12^th^ pectoral-fin rays elongate, and by 20–23% divergence in the cytochrome c oxidase I (COI) DNA barcode sequences. It further differs from one or the other of those species in head spination and in numbers of soft dorsal-fin rays, pectoral-fin rays, and precaudal + caudal vertebrae. Inhabiting depths of 95–160 m, the new species is the deepest western Atlantic member of the genus (*Scorpaenodes caribbaeus* occurs at depths < 35 m and *Scorpaenodes tredecimspinosus* from 7 to 82 m). DNA barcode data do not rigorously resolve relationships among the ten species of the genus for which those data are available.

Smithsonian’s Deep Reef Observation Project

cytochrome c oxidase I

## Introduction

Scorpionfishes of the genus *Scorpaenodes* occur circumglobally on rocky or reef substrates in tropical to temperate waters (Eschmeyer 1969, Poss and Eschmeyer 2003). Eschmeyer et al. (2016) recognize 29 valid species, 23 from the Pacific, four from the eastern Atlantic, and *Scorpaenodes caribbaeus* Meek and Hildebrand and *Scorpaenodes tredecimspinosus* (Metzelaar) from the western Atlantic. Both western Atlantic species are widely distributed, *Scorpaenodes caribbaeus* throughout the Caribbean to Brazil and north to the northern Gulf of Mexico and Bermuda, *Scorpaenodes tredecimspinosus* throughout the Caribbean to Brazil and north to North Carolina. Recent submersible diving off Curaçao in the southern Caribbean as part of the Smithsonian’s Deep Reef Observation Project (DROP) resulted in the collection of five specimens of *Scorpaenodes* that are morphologically and genetically distinct. Here we describe them as a new species and comment on depth distributions and relationships of western Atlantic members of the genus. More recent submersible collecting as part of DROP resulted in the collection of two additional specimens of the new species from Dominica Island in the eastern Caribbean.

## Methods and materials

Specimens of the new species were collected using Substation Curaçao’s (http://www.substation-Curaçao.com) manned submersible *Curasub*. The sub has two flexible, hydraulic arms, one of which is equipped with a quinaldine/ethanol-ejection system and the other with a suction hose. Anesthetized fish specimens were captured with the suction hose, which empties into a vented plexiglass cylinder attached to the outside of the sub. At the surface, the specimens were photographed, tissue sampled, and preserved. Preserved specimens were later photographed to document preserved pigment pattern and X-rayed with a digital radiography system. Counts and measurements follow Eschmeyer (1965). The last ray of both the dorsal and anal fins is split completely to the base, but each is serially supported by a single pterygiophore, and we therefore consider it a single fin ray (in contrast to Poss et al. [2010], who counted the split dorsal and anal rays as one and a half fin rays). Measurements were made weeks to months after fixation in 10% formalin and subsequent preservation in 75% ethanol and were taken to the nearest 0.1 mm with digital calipers. USNM = Smithsonian Institution National Museum of Natural History, CAS = California Academy of Sciences.

Tissue samples for DNA Barcoding were stored in saturated salt-DMSO (dimethyl sulfoxide) buffer (Seutin et al. 1991). Extraction of DNA, PCR, sequencing cytochrome c oxidase subunit I (COI), and editing COI sequences were performed as outlined by Weigt et al. (2012a). A neighbor-joining tree (Saitou and Nei 1987) was generated using PAUP*4.1 (Swofford 2002) on an analysis of Kimura two-parameter distances (Kimura 1980) for the purpose of constructing a genetic-distance table. The neighbor-joining analysis reveals genetic distances in COI among individuals and clusters them into genetically distinct lineages, which, in teleost fishes, correspond well with species (e.g. Baldwin and Weigt 2012, Weigt et al. 2012b). Interspecific phylogenetic relationships were hypothesized for western Atlantic *Scorpaenodes* and several species from other oceans (from public sequences available on GenBank) based on maximum parsimony analysis of the COI sequences using heuristic searches (100 replicates) in PAUP*4.1 (Swofford 2002). Characters were equally weighted and left unordered. The resulting equally parsimonious trees were summarized using the strict consensus method, and nodal support was estimated from 1,000 replicates of the bootstrap, utilizing random addition sequence and TBR branch swapping (Swofford 2002). The tree was rooted on *Scorpaena plumieri* Bloch, a member of the genus recovered as the sister group to *Scorpaenodes* by Smith and Wheeler (2004).

GenSeq nomenclature (Chakrabarty et al. 2013) and GenBank accession numbers for DNA sequences derived in this study are presented along with museum catalog numbers for voucher specimens in the Suppl. material 1. GenBank accession numbers for other scorpaenid sequences included in the genetic analysis are *Scorpaenodes guamensis* (Quoy and Gaimard) HQ945882, *Scorpaenodes parvipinnis* (Garrett) JQ350352, *Scorpaenodes* sp. KJ968262, *Scorpaenodes varipinnis* Smith JF494468, *Scorpaenodes kelloggi* (Jenkins) KF489747, *Scorpaenodes rubrivinctus* Poss et al. GU357570, *Scorpaenodes corallinus* Smith JQ432120, *Scorpaenodes minor* (Smith) JQ432127, and *Scorpaena plumieri* JQ8402070. BOLD accession number for *Scorpaenodes albaiensis* is DSLAF552-08.

## Taxonomy

### Stellate Scorpionfish

#### Scorpaenodes barrybrowni

Taxon classificationAnimaliaScorpaeniformesScorpaenidae

Pitassy & Baldwin sp. n.

http://zoobank.org/7511A771-86F4-46D2-8CBF-2B3C28755C94

Figs 1 , 2A

##### Type locality.

Curaçao, eastward of Substation Curaçao downline, 12.0832°N, 68.8991°W, D. R. Robertson, B. Brandt, A. Driskell, R. Loendersloot, K. Stewart, 30 May 2011.

##### Holotype.

USNM 406390, Smithsonian DNA number CUR11390, 37.1 mm SL, *Curasub* submersible, sta. 11-5, Curaçao, eastward of Substation Curaçao downline, 12.0832°N, 68.8991°W, 95–160 m, 30 May 2011, D. R. Robertson, B. Brandt, A. Driskell, R. Loendersloot, K. Stewart.

##### Paratypes.

USNM 406138, Smithsonian DNA number CUR11138, 30.4 mm SL, *Curasub* submersible, sta.11-02, Curaçao, off Substation Curaçao downline, 12.0832°N 68.8991°W, 137–146 m, 23 May 2011, C. Baldwin, D. R. Robertson, A. Schrier, B. Brandt; CAS 241446, Smithsonian DNA number CUR13257, 38.1 mm SL, *Curasub* submersible, sta. 13-14, Curaçao, off Substation Curaçao downline, 12.0832°N, 68.8991°W, 135 m, 9 August 2013, C. Baldwin, D. R. Robertson, A. Driskell, B. van Bebber; USNM 430028, Smithsonian DNA number CUR13322, 30.7 mm SL, *Curasub* submersible, sta. 13-31, Curaçao, west of Substation Curaçao downline, 12.0832°N, 68.8991°W, 223–235 m, 1 November 2013, C. Baldwin, D. R. Robertson, B. Brandt, C. Castillo; USNM 426717, Smithsonian DNA number CUR13179, 46.6 mm SL, *Curasub* submersible, Dive 2, Kralendijk, Bonaire City Dock, 12.1500°N, 68.2829°W, 114 m, 30 May 2013, C. Baldwin, A. Schrier, B. van Bebber, T. Christiaan.

##### Non-type specimens.

USNM 438436, Smithsonian DNA number DOM16034, 50.0 mm SL, *Curasub* submersible, sta. 16-11, Dominica, Prince Rupert Bay, 15.5551°N, 60.4641°W to 15.5624°N, 61.4745°W, 146 m, 7 March 2016, C. Baldwin, B. Van Bebber, A. Schrier, B. Hoeksema; USNM 438437, Smithsonian DNA number DOM16086, 45.0 mm SL, *Curasub* submersible, sta. 16-15, Dominica, Prince Rubert Bay, 15.5551°N, 61.4746°W, depth not recorded, 10 March 2016, A. Schrier, B. Van Bebber, D. Felder, A. Collins.

##### Diagnosis.

A species of *Scorpaenodes* distinguished by the following combination of characters: dorsal-fin soft rays 8; pectoral-fin rays 16–17, rays 11–12 (from uppermost ray) noticeably longer than rest in smallest four type specimens; caudal-fin rays 25–27; vertebrae 24 (8 precaudal + 16 caudal); spines on suborbital ridge 4 (rarely 5); secondary suborbital ridge spines absent; two prominent round to oblong pores in suborbital sensory canal immediately ventral to suborbital ridge; coronal, interorbital, upper post temporal and postorbital spines absent; lateral line incomplete, 8-10 pored scales extending from behind supracleithral spine to mid body; cirri associated with nasal, supraocular, and parietal spines and present on posteroventral projection of lacrimal and upper left quadrant of orbit; no cirri associated with postocular, tympanic, supracleithral, and lower posttemporal spines; body relatively elongate, depth at origin of dorsal fin 30–32% SL, depth at caudal peduncle 9–10% SL. Color in life bright orange-red with several reddish-brown bars on posterior portion of trunk; pectoral fin with vivid yellow spots interspersed with bright orange-red spots.

##### Description.

Dorsal fin XIII, 8, last soft ray split to base but supported in serial association by a single pterygiophore. Anal fin III, 5, last soft ray split to base but serially supported by single pterygiophore. Pectoral-fin rays 16–17, 17 (left)/17 (right) in holotype and three paratypes, 17/16 in one paratype. Upper-limb gill rakers 5–6 (2 rakers and 3–4 rudiments), lower limb 9–12 (8–9 rakers and 1–3 rudiments) = 14–18 total, 6 + 12 = 18 in holotype. Vertical scale rows 34–45, 41 in holotype. Pored lateral-line scales 8–10, 9 in holotype, scales extending from behind supracleithral spine to mid body. Vertebrae 8 + 16 = 24.

Morphometric data for type material given in Table 1. In the following, condition in holotype given in parentheses. Head large, length 44–48% SL (48% SL). Snout length 12–14% SL (12%), slightly shorter than orbit diameter, 14–15% SL (14%). Posterior portion of lacrimal with two somewhat rounded, ventrally directed projections. Suborbital ridge usually with 4, rarely 5, laterally directed spines (4), first at level of anterior rim of eye, second just posterior to center of eye, third and fourth posterior to orbit; spines positioned close together, with fourth spine at terminal end of suborbital ridge. Fifth spine, when present, appearing supplemental to fourth suborbital spine. Secondary suborbital ridge or spines absent. The two, large, suborbital pores positioned just below bases of second and third suborbital spines. Preopercle with 4 or 5 spines on posterior margin (5); uppermost spine largest, directed posteriorly, and in line with spines present on suborbital ridge. A conspicuous supplemental spine located immediately anterior to uppermost preopercular spine, and shafts of the two spines may appear merged with more or less distinct points; second preopercular spine sharp, located immediately ventral to first, and noticeably smaller than first and third spines; third spine more triangular in appearance, less sharp, directed posteroventrally; fourth spine similar in size or smaller than third, both directed ventrally; fifth spine rudimentary. Opercle with two pointed spines. Postocular, tympanic, parietal, nuchal, supracleithral, lower posttemporal, pterotic, and cleithral spines present, strongly developed, and with sharp points. Nasal, preocular, supraocular, and sphenotic spines distinct and pointed but diminutive relative to aforementioned spines. Interorbital ridges miniscule, lacking spines. Coronal, upper temporal, and postorbital spines absent. Cirri associated with nasal, supraocular, and parietal spines and present on posteroventral projection of lacrimal and upper left quadrant of eye. Cirri present or absent in association with preocular, nuchal, and second suborbital spines, and anteriormost of the two ventral lacrimal projections. Cirri branched or unbranched at distal tips. Supraocular cirrus noticeably longer than others. Various fleshy lappets may be present on body, especially adjacent to lateral line. No cirri on ventral surface of mandible. Anterior nostril in short tube with broad, well-developed nasal flap/cirrus on posterior margin. Posterior nostril in short tube formed posteriorly by orbit and anteriorly by sheath of transparent skin.

**Table 1.** Morphometric characters of *Scorpaenodes barrybrowni*, sp. n., expressed as percentages of standard length. Means (in parentheses) include values of the holotype.

	HOLOTYPE	PARATYPES
	USNM 406390	n = 4
Standard length (mm)	37.1	30.4–46.6
Head length	47.7	44.4–46.0 (45.9)
Snout length	12.1	11.5–13.6 (12.4)
Orbit diameter	14.0	13.5–14.5 (13.9)
Interorbital width	4.7	4.3–5.4 (4.8)
Body depth	31.0	30.0–32.2 (31.1)
Caudal peduncle depth	9.9	8.9–10.0 (9.6)
Caudal peduncle width	14.5	13.9–16.6 (14.9)
Upper jaw length	24.0	21.9–23.4 (22.9)
Pre-Pelvic length	41.0	37.7–39.8 (39.3)
Pre-Dorsal length	45.1	42.9–46.4 (44.8)
Pre-Anal length	76.3	71.5–76.2 (74.5)
First dorsal spine length	6.2	5.7–6.5 (6.1)
Second dorsal spine length	7.8	8.3–11.3 (9.8)
Longest dorsal spine length	16.2	15.2–18.1 (16.3)
Twelfth dorsal spine length	3.2	4.0–6.6 (4.6)
Thirteenth dorsal spine length	11.1	10.7–11.8 (11.2)
Longest soft dorsal ray	14.6	15.6–16.6 (15.9)
First anal spine length	9.4	8.3–10.9 (9.9)
Second anal spine length	16.4	17.4–19.8 (18.4)
Third anal spine length	14.0	14.9–15.3 (14.9)
Longest soft anal ray	18.3	17.1–20.6 (19.0)
Caudal fin length	29.4	25.7–30.1 (28.4)
Pectoral fin length	31.8	29.7–35.3 (32.9)
Pelvic spine length	18.9	17.4–19.4 (18.7)
Pelvic fin length	23.7	23.7–24.9 (24.3)

Dorsal fin originating above upper edge of opercle, fourth or fifth spines longest; penultimate shortest; fin membranes between spines incised. Anal fin with 3 spines, second longer, more robust than first or third. Uppermost pectoral-fin ray unbranched, second branched or unbranched (branched in holotype), next 7–9 rays branched (8 in holotype), ventralmost 7–8 rays unbranched (7 in holotype). Longest pectoral-fin rays usually in position 11–12 from uppermost ray and usually conspicuously longer than surrounding rays (rays in ventral half of fin broken on left side of holotype, 11^th^ and 12^th^ rays on right side conspicuously elongate). In largest type specimen (USNM 425717, 46.6 mm SL), 10^th^ pectoral-fin ray longest and 11^th^ and 12^th^ rays not distinctly longer than neighboring rays. Pectoral fin may terminate anterior to anal fin or reach past origin of anal-fin spines. Pelvic fin terminating well anterior to anal-fin insertion, pelvic spine shorter than soft pelvic rays. Caudal fin with 25–27 total rays (25), dorsal lobe with 7 unbranched + 6 branched rays, ventral lobe with 5–6 branched + 7–8 unbranched (5+7).

No prominent knob at symphysis of lower jaw. Four distinct mandibular pores, the first very small and situated immediately posterior to symphysis. Gill rakers relatively short but slender.

Pseudobranch present but with poorly formed lamellae. Premaxilla and dentary each with band of small teeth in multiple rows, bands broadest near symphysis. Vomer with chevron-shaped patch of teeth in 3–4 rows. No teeth on palatine, pterygoids, or tongue. Swimbladder present.

**Figure 1.**
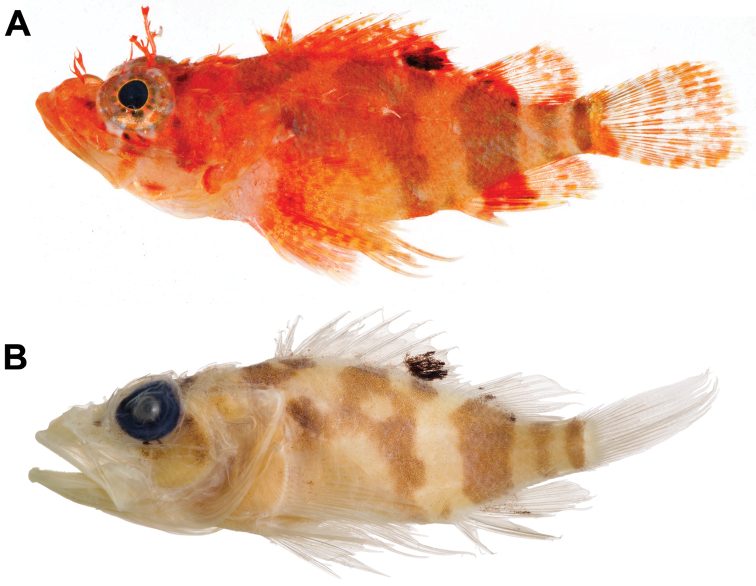
*Scorpaenodes barrybrowni* sp. n., holotype, USNM 406390, Smithsonian DNA number CUR 11390, 37.1 mm SL – before preservation (**A** photo by C. Baldwin and R. Robertson) and after preservation (**B** photo by D. Pitassy).

**Figure 2.**
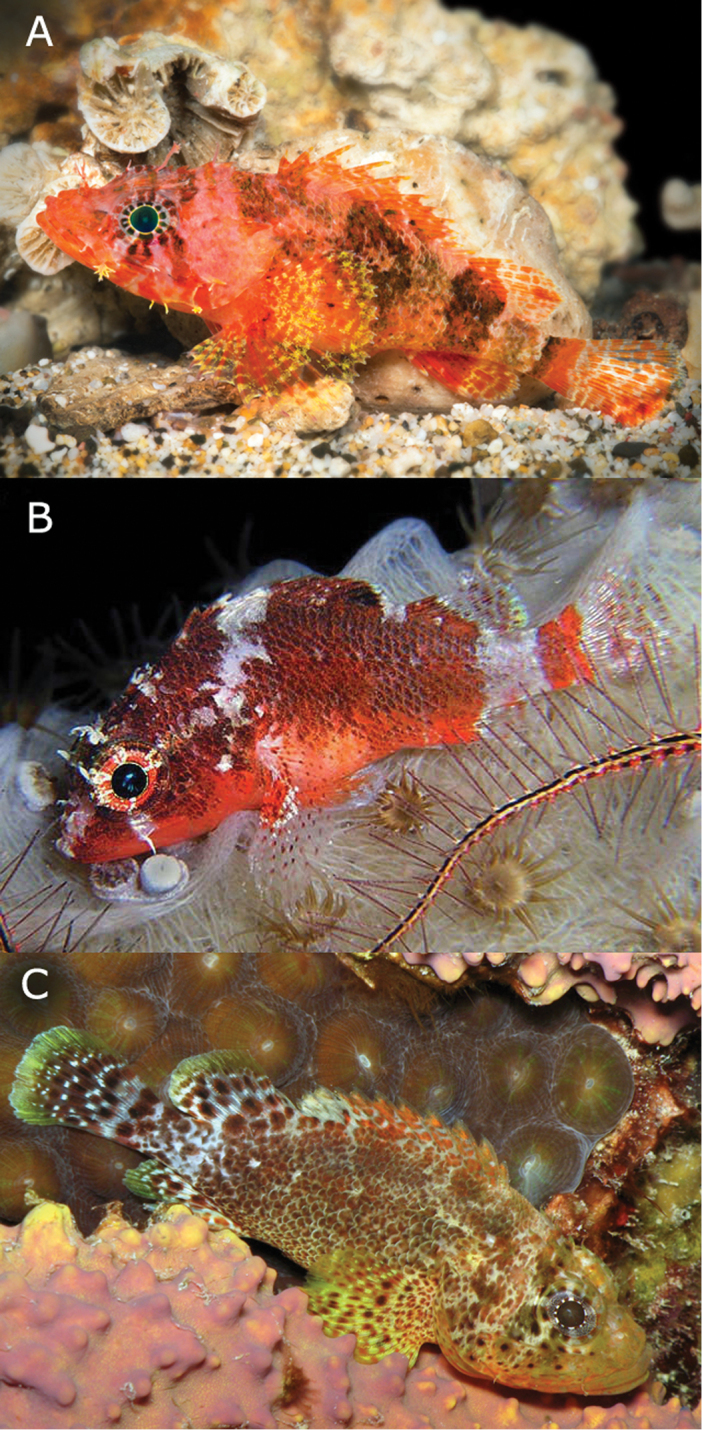
Comparison of living specimens of **A** *Scorpaenodes barrybrowni* sp. n., paratype, USNM 426717, 38.1 mm, with its two western Atlantic congeners: **B** *Scorpaenodes tredecimspinosus* and **C** *Scorpaenodes caribbaeus*. Photos: *Scorpaenodes barrybrowni* Barry Brown, *Scorpaenodes tredecimspinosus* Ellen Muller, *Scorpaenodes caribbaeus* Brian Mayes.

Color description based on image of a living specimen in an aquarium brought to the surface alive from 114 m (USNM 426717, Fig. 2A) and from color images of recently deceased type material (e.g., USNM 406390, Fig. 1A). Body mostly orange to pinkish orange, paler on underside of head and belly; lower portion of body with diffuse areas of translucent yellow pigment; body lappets pink to white. Nasal and supraorbital cirri pink to orange; pupil black, encircled successively distally by thin cream ring, ring of short dark brown or grey bars on whitish or orange background, and ring of reddish-brown to orange bars on pink to grey background; two dark markings below ventral portion of eye forming part of red/orange bar that extends ventrally across mouth; this bar followed posteriorly by short red/orange bar or marking on lower jaw; in living specimen, another two dark markings (anterior one part of outer orbital ring of pigment) forming part of oblique, reddish-brown bar extending from posteroventral portion of orbit to posteroventral edge of operculum; snout, anterior portions of jaws, and dorsal portion of cheek pink to orange; posterior portions of jaws and ventral portion of remainder of head pink to clear; series of indistinct, narrow reddish-brown bars present across dorsal portions of head and nape; lappets on jaws creamy yellow. Body with five orange-brown to brown blotches, posteriormost three forming well-defined bars: anteriormost blotch on nape above rear corner of opercle and extending posteriorly beneath first three dorsal-fin spines; second blotch broadest, irregular in shape, with pale center, situated mostly beneath dorsal spines 6-10 and narrowing ventrally but extending anteriorly to rear edge of opercle along lateral line; third marking a bar beneath anterior half of soft dorsal fin; fourth the narrowest and most indistinct, located on caudal peduncle immediately behind bases of dorsal and anal fins; and fifth strong, narrow, and situated on posterior edge of caudal peduncle. Spinous dorsal fin mostly orange with scattered pale or pale pink streaks and usually a blackish-brown ovoid blotch across bases of spines 7–10; this dark blotch poorly defined in 46.6-mm SL specimen (USNM 426717, Fig. 2A) and appearing as several smaller spots; soft-dorsal fin with broad, bright orange stripe at base, sometimes with a few dark spots near the base; remainder of fin mainly clear with two to several irregular rows of orange spots; smallest specimen (30.4 mm SL) without dark body blotches or bars and without broad stripe of orange at base of soft dorsal fin. Base of caudal fin with strong orange bar immediately behind posteriormost peduncular bar, rest of fin with translucent membranes and irregular rows of elongate orange spots. Anal fin with broad orange stripe at base, distal portion of fin with translucent membranes and 2–3 irregular rows of orange spots; orange stripes at bases of soft dorsal and anal fins connected to third body blotch (anteriormost of the three well-defined bars), giving the appearance of a single orange (fins) to orangish-brown (body) bar. Pelvic fin spine whitish, basal half of soft-pelvic fin pale orange, fading distally to translucent/yellowish. Pectoral fin base pale pinkish, base of fin orange, orange extending posteriorly along several rays in ventral portion of fin; remainder of fin paler and with irregular pattern of orange and yellow spots; yellow xanthophores appearing as expanded, stellate pigment markings in living specimen (Fig. 2A).

Color of holotype in alcohol (Fig. 1B). Ground color light tan. Head with dark grey eye with varying numbers of short, radiating, dark brown lines or bars, some of these extending onto portions of head adjacent to orbit; a dark blotch beneath eye above posterior end of upper jaw; another dark blotch extending from posterior edge of orbit obliquely to rear edge of preopercle; snout, maxilla, operculum, and nape with scattered dark spots, a dark blotch above posterodorsal region of orbit. Body with five dark blotches or bars described in fresh specimens retained in preservative. Dorsal fin translucent with scattered dark specks and an oval black blotch on bases of spines 7-10 in all type specimens except the largest (46.6 mm SL), which has scattered dark markings on spines 6-10 as well as a bit of dark pigment at bases of spines 1-3; soft dorsal with dark blotch at base above dark body bar; remainder of fin clear. Caudal fin translucent. Anal fin translucent, usually with dark smudge on fin continuous with dark body bar. Pelvic fins translucent. Pectoral fins translucent, with one or more irregular dark spots on central fin rays.

##### Distribution.

Known from Curaçao and Bonaire in the southern Caribbean, and Dominica in the Windward Islands, eastern Caribbean.

##### Habitat.

Collected off Curaçao at 95–160 m on rocky substrata. Off Dominica, USNM 438436 was collected on a vertical rock wall.

##### Etymology.

Named in honor of Barry Brown, Substation Curaçao and free-lance photographer (www.coralreefphotos.com), who has patiently, diligently, and expertly taken photographs of hundreds of fishes and invertebrates captured alive by DROP investigators. He has generously shared his photographs, and they have enhanced numerous scientific and educational publications. An example of his work is here featured in Fig. 2A.

##### Common name.

Stellate Scorpionfish, in reference to the yellow, stellate chromatophores on the pectoral fin in life and the radiating pigment markings accentuating the eye. Spanish common name: Escorpión Estrellado.

##### Genetic comparisons.

**Figure 3.**
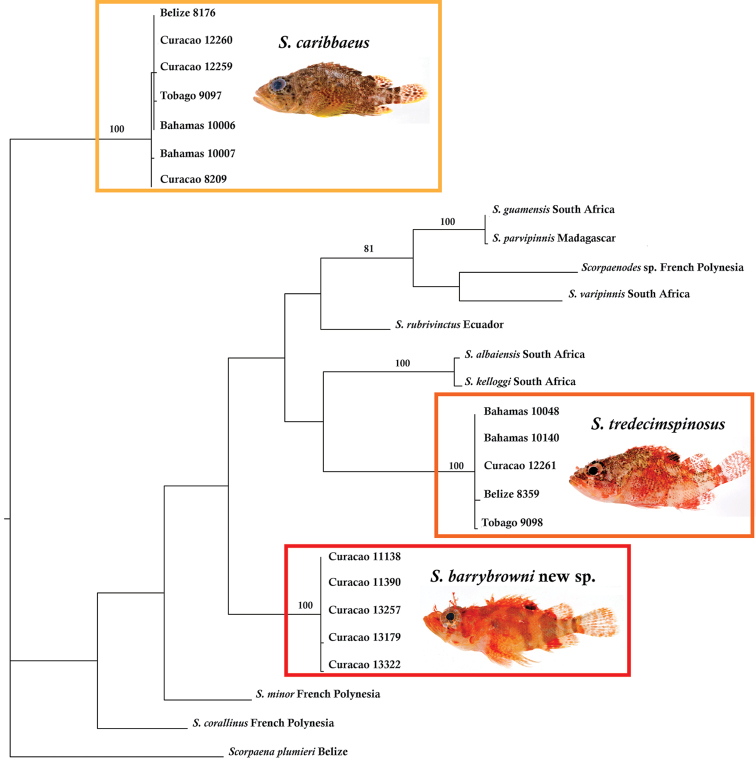
The strict consensus of a maximum parsimony analysis of the COI region of 26 individuals of *Scorpaenodes*. The tree was rooted on *Scorpaena plumieri*. Numbers above branches represent bootstrap support values > 50.

Figure 3 shows the results of the maximum parsimony analysis of the COI sequences, which clearly support recognizing *Scorpaenodes barrybrowni* as a species distinct from western Atlantic *Scorpaenodes caribbaeus* and *Scorpaenodes tredecimspinosus*. Table 2 shows genetic distances within each species and between pairs of species included in the analysis. Intraspecific genetic variation is 0.0–0.2% for *Scorpaenodes barrybrowni* and 0.0–0.5% for both *Scorpaenodes caribbaeus* and *Scorpaenodes tredecimspinosus*, whereas interspecific divergences among the ten members of the genus for which data are available, including *Scorpaenodes barrybrowni*, are 14.5–23.2%. The COI data are insufficient to resolve most relationships among *Scorpaenodes* species with any confidence (only bootstrap values >50 are shown on the tree). A clade comprising *Scorpaenodes guamensis*, *Scorpaenodes parvipinnis*, *Scorpaenodes varipinnis*, and an unidentified *Scorpaenodes* from French Polynesia has a bootstrap value of 81. Note that *Scorpaenodes guamensis* from South Africa and *Scorpaenodes parvipinnis* from Madagascar appear to be the same species (0.2% divergence), which either indicates that they are synonymous or one of the specimens from which the sequences in GenBank were derived is misidentified. Likewise, *Scorpaenodes albaiensis* and *Scorpaenodes kelloggi* from South Africa are very similar (0.8% divergence). If one constructs a neighbor-joining tree online at BOLD (http://www.boldsystems.org/) for *Scorpaenodes*, there are numerous misidentifications or taxonomic issues that need to be resolved. For example, *Scorpaenodes varipinnis*, *Scorpaenodes parvipinnis*, and *Scorpaenodes guamensis* all appear in at least three genetic lineages. Additional analyses are needed, but our preliminary COI data would not appear to support a monophyletic clade of western Atlantic *Scorpaenodes*.

**Table 2.** Range and average Kimura two-parameter distance summary for species of *Scorpaenodes* based on cytochrome c oxidase I (COI) sequences analyzed genetically in this study. Intraspecific values are in bold.

	*Scorpaenodes caribbaeus*	*Scorpaenodes guamensis*	*Scorpaenodes parvipinnis*	*Scorpaenodes* sp.	*Scorpaenodes varipinnis*	*Scorpaenodes barrybrowni* sp. n.	*Scorpaenodes albaiensis*	*Scorpaenodes kelloggi*	*Scorpaenodes rubrivinctus*	*Scorpaenodes corallinus*	*Scorpaenodes minor*	*Scorpaenodes tredecimspinosus*
*Scorpaenodes caribbaeus*	**0.0–0.5 (0.2)**											
*Scorpaenodes guamensis*	20.0–20.3 (20.2)	**NA**										
*Scorpaenodes parvipinnis*	20.1–20.3 (20.2)	0,2	**NA**									
*Scorpaenodes* sp.	21.8–22.0 (21.9)	15,1	15,1	**NA**								
*Scorpaenodes varipinnis*	19.4–19.8 (19.6)	14,3	14,3	14,8	**NA**							
*Scorpaenodes barrybrowni*	21.5–23.2 (21.8)	15.5–15.7 (15.6)	15.2–15.3 (15.3)	19.6–19.8 (19.7)	19.5–19.7 (19.6)	**0.0–0.2 (0.1)**						
*Scorpaenodes albaiensis*	18.3–18.5 (18.4)	18,8	18,5	20,7	18,2	16.3–16.5 (16.4)	**NA**					
*Scorpaenodes kelloggi*	18.7–19.1 (18.9)	18,6	18,3	20,9	18,6	16.1–16.3 (16.2)	0,8	**NA**				
*Scorpaenodes rubrivinctus*	18.7–19.0 (18.9)	14,7	14,5	18,5	15,9	14.5–14.7 (14.6)	14,7	14,9	**NA**			
*Scorpaenodes corallinus*	18.5–18.9 (18.7)	19,3	19,5	20,5	18,4	16.3–16.5 (16.4)	21,5	21,7	19,2	**NA**		
*Scorpaenodes minor*	21.1–21.6 (21.3)	17,2	17,4	18,4	17,7	15.1–15.3 (15.2)	17,2	0,2	15,4	15,6	**NA**	
*Scorpaenodes tredecimspinosus*	18.8–19.7 (19.2)	17.5–18.1 (17.6)	17.7–18.3 (17.8)	18.7–19.0 (18.9)	20.2–20.4 (20.2)	18.4–19.0 (18.8)	19.4–19.7 (19.7)	19.8–20.3 (20.1)	18.2–18.6 (18.3)	18.8–19.4 (19.0)	19.9–20.2 (19.0)	**0.0–0.5 (0.2)**

##### Morphological comparisons.

The presence of thirteen dorsal-fin spines and absence of palatine teeth support the placement of the new species in the genus *Scorpaenodes* (Poss and Eschmeyer 2003). The combination of features provided in the diagnosis distinguishes *Scorpaenodes barrybrowni* from all congeners. Characters that distinguish *Scorpaenodes barrybrowni* from one or both of its western Atlantic congeners, *Scorpaenodes caribbaeus* and *Scorpaenodes tredecimspinosus*, are tabulated in Table 3 and summarized below. *Scorpaenodes barrybrowni* usually has a shallower body (depth at dorsal-fin origin 30–32% SL vs. 32–41% SL in *Scorpaenodes caribbaeus* and *Scorpaenodes tredecimspinosus*), usually a shallower caudal peduncle (depth 9–10% SL vs. 10–12% SL), two large round pores below the suborbital ridge (vs. several small pores), an incomplete lateral line comprising 8–10 pored scales (vs. a complete lateral line comprising 22–25 pored scales), and more caudal-fin rays (25–27 vs. usually 23–24). Interorbital, coronal, and upper posttemporal spines were not observed in *Scorpaenodes barrybrowni*, but all three are generally present in *Scorpaenodes caribbaeus* and the interorbital and coronal (and sometimes the upper posttemporal) are present in *Scorpaenodes tredecimspinosus*. Furthermore, *Scorpaenodes barrybrowni* has more slender gill rakers than the short, stubby elements of *Scorpaenodes caribbaeus* and *Scorpaenodes tredecimspinosus*; and the pseudobranch of *Scorpaenodes barrybrowni* is poorly formed, with fat, sausage-like lamellae vs. a very well-organized, comb-like pseudobranch in *Scorpaenodes caribbaeus* and *Scorpaenodes tredecimspinosus*. Color pattern of *Scorpaenodes barrybrowni* is distinct from that of *Scorpaenodes caribbaeus* and *Scorpaenodes tredecimspinosus* in many aspects (Fig. 2), but notably from the former in generally being bright orange (vs. mostly brown to reddish-brown) and from the latter in having yellow pigment on the pectoral fin. Finally, *Scorpaenodes barrybrowni* inhabits depths of 95–160 m vs. < 35 m for *Scorpaenodes caribbaeus* and 8–82 m for *Scorpaenodes tredecimspinosus* (Robertson and Van Tassell 2015).

**Table 3.** Comparison of morphological characters in *Scorpaenodes barrybrowni*, sp. n., *Scorpaenodes caribbaeus*, and *Scorpaenodes tredecimspinosus*.

	*Scorpaenodes barrybrowni* sp. n. n = 5	*Scorpaenodes caribbaeus* n = 15	*Scorpaenodes tredecimspinosus* n = 13
Maximum SL (mm)	< 50	> 60	< 50
Body depth/ SL	30–32%	33–39%	32–41%
Caudal peduncle depth/ SL	9–10%	10–12%	11–12%
Soft dorsal-fin rays	8	9	9
Pectoral-fin rays	16–17	18–20	16–17
Caudal-fin rays	25–27	23–24	23–24
Pre-caudal + caudal vertebrae	8+16	9+15	8+16
Pored lateral line scales	8–10	22–25	22–23
Suborbital spines	4–5	3–6	1–3
Secondary suborbital spines	Absent	Present	Absent
Suborbital ridge pores	2, large	Multiple, small	Multiple, small
Coronal spines	Absent	Usually present	Usually present
Interorbital spines	Absent	Usually present	Usually present
Upper posttemporal spines	Absent	Usually present	Sometimes present
Gill raker shape	Slender	Stout	Stout
Pseudobranch lamallae	Stout	Slender	Slender
Pectoral fin color	Orange-red and yellow	Brownish red and yellow	Dark red and pink

Additional characters that differentiate *Scorpaenodes barrybrowni* from *Scorpaenodes caribbaeus* include fewer soft dorsal-fin rays (8 in *Scorpaenodes barrybrowni* vs. 9 in *Scorpaenodes caribbaeus*); usually fewer pectoral-fin rays (16–17 vs. 17–20); absence of secondary suborbital spines (vs. usually 1 or more), different vertebral counts (8 precaudal + 16 caudal vs. 9 + 15), fewer spine-associated cirri on head (*Scorpaenodes barrybrowni* lacks cirri associated with postocular, tympanic, supraclethral, and lower posttemporal spines, *Scorpaenodes caribbaeus* has cirri associated with most spines on the head). *Scorpaenodes barrybrowni* is smaller, reaching approximately 47 mm SL vs. 85 mm SL in *Scorpaenodes caribbaeus*.

Additional characters that distinguish *Scorpaenodes barrybrowni* from *Scorpaenodes tredecimspinosus* include more suborbital spines (4–5 in *Scorpaenodes barrybrowni* vs. usually 2, occasionally 1 or 3, in *Scorpaenodes tredecimspinosus*) and fewer soft dorsal-fin rays (8 vs. 9). Both species reach a similar maximum size (47 vs. 45 mm SL).

##### Comparative material examined.

*Scorpaenodes caribbaeus*, 15 specimens, 20.1–63.7 mm. BAHAMAS: USNM 415441, Smithsonian DNA number BAH 10006, 43.3 mm SL, BAH 10-01, Berry Islands, Great Stirrup Cay, 25.8261°N, 77.9189°W, 6–9 m, 7 August 2010, C. Baldwin, A. Driskell, L. Lang; USNM 415442, Smithsonian DNA number BAH 10007, 29.4 mm SL, BAH 10-01, Berry Islands, Great Stirrup Cay, 25.8261°N, 77.9189°W, 6–9 m, 7 August 2010, C. Baldwin, A. Driskell, L. Lang. BELIZE: USNM 404029, Smithsonian DNA number BLZ10029, 25.5 mm SL, CB10-02, Shallow spur and groove off north side of Carrie Bow Cay, 16.8007°N, 88.0783°W, 0–12 m, 11 November 2010, C. Baldwin, M. Fagan-Halloran; USNM 415314, Smithsonian DNA number BLZ 8313, 27.1 mm SL, CB 08-29, Sand bores ~ 3 miles southwest of Carrie Bow Cay, 16.7718°N, 88.1117°W, 0–9 m, 24 May 2008, C. Baldwin, Z. Foltz, D. Smith; USNM 415248, Smithsonian DNA number BLZ 8176, 32.9 mm SL, CB 08-17, Whale Shoals, South Cut, in and out of reef, 16.7598°N, 88.0761°W, 0–5 m, 20 May 2008, C. Baldwin, Z. Foltz, L. Weigt; USNM 415016, Smithsonian DNA number BLZ 7156, 33.5 mm SL, CB 07-14, Outer ridge east of Carrie Bow Cay, 21–23 m, 16 January 2007, D. Miller, J. Mounts; USNM 421926, Smithsonian DNA number BLZ 8358, 48.1 mm SL, CB 08-32, Tobacco Cay, 16.8899°N, 88.0649°W, 0–5 m, 25 May 2008, C. Baldwin, Z. Foltz, D. Smith, L. Weigt. CURAÇAO: USNM 413616, Smithsonian DNA number CUR 8209, 46.8 mm SL, CUR 08-04, Boca Sami, 12.1487°N, 68.9994°W, 0–3 m, 13 March 2008, C. Baldwin, L. Weigt; USNM 414799, Smithsonian DNA number CUR 12259, 25.6 mm SL, CUR12-03, Klein Curaçao, northwest tip of island, 6–15 m, 11 August 2012, C. Baldwin, A. Driskell; USNM 413818, Smithsonian DNA number CUR 12260, 20.1 mm SL, CUR12-03, Klein Curaçao, northwest tip of island, 6–15 m, 11 August 2012, C. Baldwin, A. Driskell. PANAMA: USNM 81619, Holotype, 63.7 mm SL, Toro Point, Canal Zone, Atlantic at Colon, 19 May 1911, S. Meek, S. Hildebrand. TRINIDAD AND TOBAGO: USNM 413274, Smithsonian DNA number Smithsonian DNA number TOB 9097, 50.8 mm SL, TOB 09-04, Tobago, Store Bay, 11.1558°N, 60.8423°W, 5–9 m, 16 March 2009, C. Baldwin, L. Weigt, D. Smith; USNM 413273, Smithsonian DNA number TOB 9096, 55.1 mm SL, TOB 09-04, Tobago, Store Bay, 11.1558°N, 60.8423°W, 5–9 m, 16 March 2009, C. Baldwin, L. Weigt, D. Smith. TURKS AND CAICOS ISLANDS: USNM 414116, Smithsonian DNA number TCI 9394, 41 mm SL, TCI 09-09, South Caicos, East Bay, 21.5374°N, 71.4801°W, 0–5 m, 9 October 2009, J. Williams, C. Castillo, M. Fagan-Halloran, B. Holt, B. Matulis; USNM 414115, Smithsonian DNA number TCI 9393, 45 mm SL, TCI 09-09, South Caicos, East Bay, 21.5374°N, 71.4801°W, 0–5 m, 9 October 2009, J. Williams, C. Castillo, M. Fagan-Halloran, B. Holt, B. Matulis.

*Scorpaenodes tredecimspinosus*, 13 specimens, 20.7–44.8 mm. BAHAMAS: USNM 415463, Smithsonian DNA number BAH 10048, 35.3 mm SL, BAH 10-07, Berry Islands, Great Stirrup Cay, 25.8261°N, 77.9189°W, 9 m, 9 August 2010, C. Baldwin, A. Driskell, J. Lang; USNM 415512, Smithsonian DNA number BAH 10140, 26.7 mm SL, BAH 10-11, Berry Islands, Chub Cay, 25.3993°N, 77.8909°W, 21–24 m, 11 August 2010, C. Baldwin, A. Driskell. BELIZE: USNM 415200, Smithsonian DNA number BLZ 8037, 24.7 mm SL, CB 08-02, Curlew, outer ridge, 16.7900°N, 88.0781°W, 5–8 m, 15 May 2008, C. Baldwin, Z. Foltz, L. Weigt; USNM 415331, BLZ 8359, 29.3 mm SL, CB 08-32, Tobacco Cay, 16.8899°N, 88.0649°W, 0–5 m, 25 May 2008, C. Baldwin, Z. Foltz, D. Smith, L. Weigt. BONAIRE: USNM 216451, Paralectotype, 36.1 mm SL, Dutch West Indies, Bonaire, 1904, J. Boeke. CURAÇAO: USNM 413408, Smithsonian DNA number CUR 8204, 28.1 mm SL, CUR 08-04, Boca Sami, 12.1487°N, 68.9994°W, 0–3 m, 13 March 2008, C. Baldwin, L. Weigt; USNM 413812, Smithsonian DNA number CUR 12261, 25.3 mm SL, CUR12-03, Klein Curaçao, northwest tip of island, 11.9985°N, 68.6513°W, 6–15 m, 11 August 2012, C. Baldwin, A. Driskell; USNM 413836, Smithsonian DNA number CUR 12178, 20.7 mm SL, CUR12-02, Klein Curaçao, southwest tip of island, 11.9758°N, 68.6462°W, 0–3 m, 11 August 2012, D. R. Robertson, C. Castillo, P. Mace. TRINIDAD AND TOBAGO: USNM 319121, 37.7 mm SL, JTW 90-10, Tobago, Buccoo Reef, outer reef slope, 11.1850°N, 60.8228°W, 14 m, 10 September 1990, J. Williams, J. Howe, G. Johnson, S. Blum, M. Nizinski, T. Munroe; USNM 413271, Smithsonian DNA number TOB 9098, 33.7 mm SL, TOB 09-04, Tobago, Store Bay, 11.1558°N, 60.8423°W, 5–9 m, 16 March 2009, C. Baldwin, L. Weigt, D. Smith. TURKS AND CAICOS ISLANDS: USNM 411912, Smithsonian DNA number TCI 9036, 22.8 mm SL, TCI 09-01, South Caicos, East Bay, 21.4919°N, 71.5176°W, 0–2 m, 7 October 2009, C. Baldwin, J. Williams, L. Weigt, C. Castillo, M. Fagan-Halloran, B. Holt, B. Matulis. UNITED STATES, FLORIDA: USNM 108875, 38.3 mm SL, South of Tortugas, 82 m, 14 July 1915, W. Longley; USNM 108876, 44.8 mm SL, South of Tortugas, 82 m, 14 July 1915, W. Longley.

## Discussion

Poss et al. (2010) noted that the limits of *Scorpaenodes* are uncertain. Historically, shallow-water species such as *Scorpaenodes albaiensis* (Evermann and Seale) and *Scorpaenodes minor* (Smith), in which the uppermost unbranched rays of the pectoral fin are elongate, have been placed in *Hypomacrus*. Eschmeyer (1969) relegated *Hypomacrus* to the synonymy of *Scorpaenodes*, Mandrytsa (2001) recognized *Hypomacrus* as valid, and Poss et al. (2010) followed Eschmeyer’s classification. We tentatively follow Eschmeyer (1969) and Poss et al. (2010) in placing the new species in *Scorpaenodes* but note that the smallest four type specimens of *Scorpaenodes barrybrowni* (30.4–38.1 mm SL) have the uppermost unbranched rays of the pectoral fin (11^th^ and 12^th^ from the top) elongate. The largest type specimen, 46.6 mm SL, lacks elongate pectoral-fin rays, but the two specimens from Dominica are large (45.0 and 50.0 mm SL) and have the 11^th^ and 12^th^ rays elongate. Further study is needed to determine if factors other than evolutionary history influence this morphological character.

*Scorpaenodes barrybrowni* is the ninth new fish species described from deep reefs of the southern Caribbean and discovered through manned submersible diving as part of the Smithsonian’s Deep Reef Observation Project – DROP (Baldwin and Robertson 2013, 2014, 2015; Baldwin and Johnson 2014; Tornabene et al. 2016a). The new species range in depth from 70–240 m, and they all belong to genera that also comprise species inhabiting shallower reef depths. Relationships between shallow- and deep-reef congeners are poorly understood, as scarce access to or no knowledge of the deep-reef species has hindered inclusive phylogenetic analyses. In a recent molecular phylogenetic analysis incorporating new deep-reef goby species from the southern Caribbean, Tornabene et al. (2016b) found multiple, co-occurring but independent transitions from shallow to deep reefs with subsequent species radiations on deep reefs in some genera. Considerably more molecular data and better taxon sampling are needed to conduct similar investigations of depth transitions in *Scorpaenodes*.

Numerous other new fish and invertebrate species already discovered through exploratory submersible diving by DROP await description, and ongoing submersible diving in the southern and other parts of the Caribbean will almost certainly result in the continued discovery of new marine life. Globally, tropical deep reefs, which are below depths accessible with conventional scuba gear and above depths typically frequented by deep-diving submersibles, are diverse, underexplored ecosystems.

## Supplementary Material

XML Treatment for Scorpaenodes barrybrowni

## Appendix

**Table:** Table Links between DNA voucher specimens, GenBank accession numbers, and cytochrome c oxidase subunit I (COI) sequences of *Scorpaenodes* derived for use in this study. Other GenBank accession information is provided in the Materials and methods section. CUR = Curaçao, BAH = Bahamas.

Catalog Number/DNA Number	GenBank No.	GenSeq Designation
*Scorpaenodes barrybrowni* n. sp.		
USNM 406390, CUR 11390, Holotype	KX419779	genseq-1 COI
USNM 406138, CUR 11138, Paratype	KX419778	genseq-2 COI
CAS 241446, CUR 13257, Paratype	KX459119	genseq-2 COI
USNM 430028, CUR 13322, Paratype	KX459120	genseq-2 COI
USNM 426717, CUR 13179, Paratype	KX459118	genseq-2 COI
*Scorpaenodes tredecimspinosus*		
USNM 415463, BAH 10048	KX419789	genseq-4 COI
USNM 415512, BAH 10140	KX419786	genseq-4 COI
USNM 413812, CUR 12261	KX419788	genseq-4 COI
*Scorpaenodes caribbaeus*		
USNM 415441, BAH 10006	KX419783	genseq-4 COI
USNM 415442, BAH 10007	KX419785	genseq-4 COI
USNM 414799, CUR 12259	KX419782	genseq-4 COI
USNM 413818, CUR 12260	KX419781	genseq-4 COI
